# Efficacy and safety of skin-adhesive low-level light therapy for overactive bladder: a Phase III study

**DOI:** 10.1007/s00192-022-05153-1

**Published:** 2022-04-07

**Authors:** Woo Yeon Hwang, Yong Beom Kim, Sa Ra Lee, Dong Hoon Suh, Kidong Kim, Jae Hong No

**Affiliations:** 1 Department of Obstetrics and Gynecology, Seoul National University College of Medicine, Seoul, Republic of Korea; 2 Department of Obstetrics and Gynecology, Seoul National University Bundang Hospital, 82 Gumi-ro 173 Beon-gil, Bundang-gu, Seongnam-si, Gyeonggi-do 13620 Republic of Korea; 3 Department of Obstetrics and Gynecology, Asan Medical Center, University of Ulsan College of Medicine, Seoul, Republic of Korea

**Keywords:** Low-level light therapy, Overactive bladder, Randomized controlled trial

## Abstract

**Introduction and hypothesis:**

Overactive bladder (OAB) is a common condition that remains challenging to treat. We hypothesized that skin-adhesive low-level light therapy (LLLT) would be an effective treatment for OAB caused by bladder muscle contraction. Accordingly, we aimed to evaluate the efficacy and safety of an LLLT device for the treatment of OAB.

**Methods:**

This prospective, randomized, double-blind, placebo-controlled, multicenter trial included patients with a clinical diagnosis of OAB who were treated at either of two university hospitals. Patients were instructed to apply an LLLT device (Color DNA-WSF) or a sham device at home three times daily for 12 weeks. The primary outcome was the change in the mean daily number of urge urinary incontinence (UUI) episodes between baseline and 12 weeks. The secondary outcomes were the mean changes in incontinence, voiding, and nocturia episodes from baseline and the likelihood of achieving a > 50% reduction in UUI and incontinence episodes after 12 weeks. All patients completed the Overactive Bladder Symptom Score (OABSS), Urogenital Distress Inventory-6 (UDI-6), and Impact Urinary Incontinence-7 (IIQ-7) questionnaires. Safety parameters included treatment-emergent adverse events.

**Results:**

Compared with those in the sham group, the numbers of UUI and urinary incontinence episodes in the LLLT group were significantly decreased at week 12 (UUI, (-1.0 ± 1.7 vs. -0.4 ± 2.5, *P* = 0.003; urinary incontinence, -1.1 ± 1.9 vs. -0.5 ± 2.9, *P*=0.002). Furthermore, the OABSS, UDI-6, and IIQ-7 scores at week 12 tended to be better in the LLLT group than in the sham group. The incidence of device-related treatment-emergent adverse events was similar between groups.

**Conclusions:**

LLLT may be clinically useful and safe for the treatment of OAB.

## Introduction

Overactive bladder (OAB) is a syndrome that manifests as lower urinary tract dysfunction and is defined as urinary urgency with or without urge incontinence and usually with frequency and nocturia in the absence of any underlying pathologic condition [1, 2]. The estimated prevalence of OAB ranges from 10.8% to 18.4% in studies from Europe, the US, and Korea [3–5]. Conventional treatment options for OAB include behavioral therapy and pharmacologic agents, including oral antimuscarinics and β_3_-adrenoceptor agonists [6]. However, although pharmacologic therapies are often effective, they are discontinued in many cases because of bothersome adverse effects and compliance problems [7]. Therefore, non-pharmacologic therapy may be a viable option to minimize symptoms and enhance the quality of life of patients with OAB.

The potential benefits of low-level light therapy (LLLT), which is delivered by light-emitting diodes, have been attracting interest in various clinical fields [8]. LLLT has been used mainly for relief of muscle pain, and a randomized controlled study demonstrated it to be an effective and safe treatment option for primary dysmenorrhea [9, 10]. Given the known efficacy of LLLT in relief of pain caused by contraction of uterine smooth muscle, we hypothesized that it might also be effective in relieving symptoms of OAB caused by involuntary muscle contractions in the bladder. Previous research has demonstrated that smooth muscle cells typically contain light receptors [11]. LLLT increases the concentration of cyclic adenosine monophosphate, which causes relaxation of smooth muscles [9, 12], and also induces nitric oxide (NO), which acts as a vasodilator and improves blood circulation [13].

In this study, we used the device known as the Woman Stress-Free (Color DNA-WSF; Color Seven Co., Seoul, Korea), which consists of a power supply unit and two microprocessor-controlled light-emitting diodes which direct the LLLT to two acupuncture points. We selected two of the acupuncture points often used to treat OAB, namely, conception vessel (CV) 6 (Qihai) and CV4 (Guanyuan) [14]. We hypothesized that a skin-adhesive LLLT device applied at two acupuncture points might be an effective treatment for OAB. The aim of this study was to assess the efficacy and safety of an LLLT device used to treat patients with OAB.

## Materials and methods

### Study design and patients

This multicenter, randomized, double-blind, placebo-controlled trial included patients aged 20 years or older with symptoms of OAB who were enrolled between March 2017 and February 2020. All patients were diagnosed by symptoms defined as at least one episodes of urgency with or without urinary incontinence per 24-h period associated with frequency and nocturia based on the patient history taking. The following exclusion criteria were applied: presence of urinary infection or urinary stones; previous surgical treatment for urinary incontinence; a large pelvic mass (e.g., myoma, adenomyoma, endometriosis) that could affect urination; previous pelvic irradiation; previous diagnosis of an invasive tumor in the pelvis; dysmenorrhea or other low abdominal symptoms; participation in another clinical study within the previous 3 months; administration of medication for OAB within the previous 3 months; pregnancy. Treatment discontinuation criteria were as follows: investigator judgement; participant voluntary withdrawal; either treatment related or unrelated toxicity; noncompliance.

After enrollment, eligible patients were randomly assigned to a sham (control) group or an LLLT (experimental) group in a 1:1 ratio. Block randomization stratified by center was performed by an independent research statistician. Patients were randomized to the LLLT group or the sham group before the start of the study using computer-generated random numbers. The sham device works in the same way as the active device but an aperture is blocked from emitting light. Researchers and patients did not know whether the light was irradiated or not. Except for the clinical research coordinator who provided a color DNA-WSF or placebo device to each patient depending on their group allocations, both patients and investigators were blinded to treatment assignment.

**Fig. 1.**
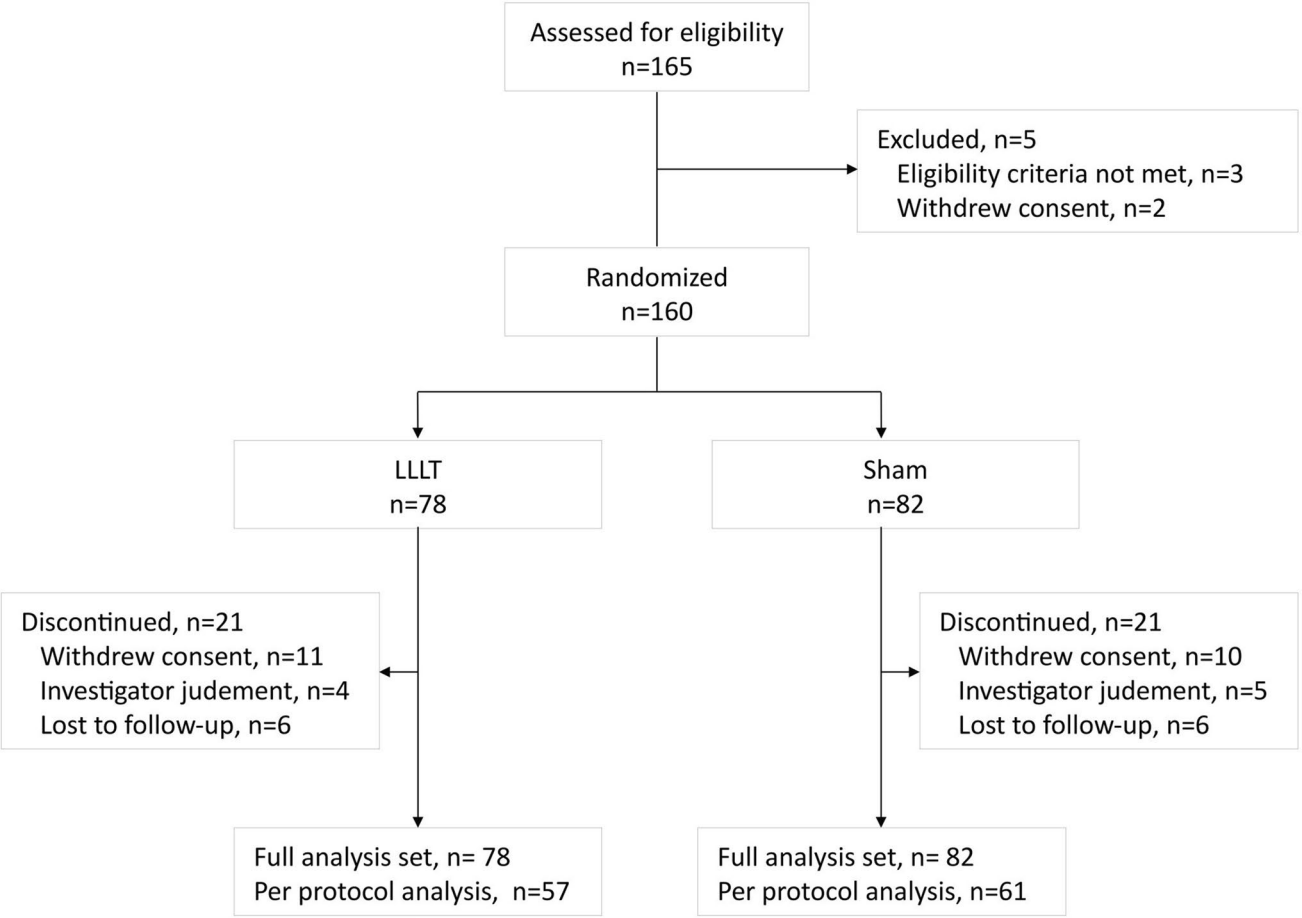
Flow of patients through the study

The study was approved by the institutional review board of each participating center and was conducted in accordance with the International Conference on Harmonization of Good Clinical Practice guidelines and the Declaration of Helsinki. All study participants signed an informed consent document after receiving adequate information about the study before its initiation. The trial is registered at ClinicalTrials.gov (NCT02980328) (Fig. 1).

### Intervention

**Fig. 2.**
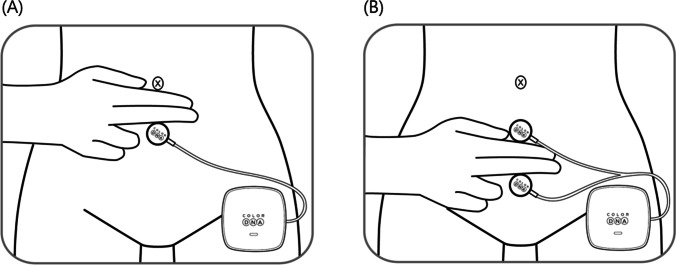
Placement of low-level light therapy probes at conception vessel 4 (**A**) and conception vessel 6 (**B**)

LLLT was self-performed three times daily for 20 min per session for 12 weeks. We used a skin-adhesive LLLT device called the Color DNA-WSF, a class II device consisting of a power supply unit and two microprocessor-controlled light-emitting diodes. An adhesive light-emitting diode light source had peak wavelength 610 ± 10 nm, light-output intensity 1.8 mW/cm^2^ ± 20%, power consumption 2.5 W ± 10% and rated current 300 mA ± 10%. Patients in both studies attached the skin-adhesive LLLT device probes to the two acupuncture points according to the treatment schedule. We selected two of the acupuncture points often used to treat OAB, namely, CV6 (Qihai), approximately two fingerbreadths inferior to the umbilicus, and CV4 (Guanyuan), approximately two fingerbreadths inferior to CV4 (Fig. 2). The sham and LLLT devices were identical in appearance except that the central hole in the sham device was blocked to prevent emission of light. In this way, the patients in the study could be blinded to their treatment allocation. All subjects were asked to fill out a diary to annotate the application to evaluate whether the medical device was used properly.

### Efficacy and safety assessments

Efficacy was assessed by asking patients to complete a 3-day voiding diary that included all symptoms of OAB, including episodes of voiding, urgency, incontinence, and nocturia before each scheduled visit at baseline and after 4 and 12 weeks of treatment. They also completed the Overactive Bladder Symptom Score (OABSS) questionnaire, which evaluates symptoms of nocturia, urgency, and urge incontinence at baseline and after 4 and 12 weeks of treatment. The OABSS comprises four items related to OAB symptoms, namely daytime frequency, nighttime frequency, urgency, and UUI. The total score ranges from 0 to 15, and a higher score represents greater symptom severity. All patients also completed the Urogenital Distress Inventory-6 (UDI-6) and Impact Urinary Incontinence-7 (IIQ-7) questionnaires at baseline and weeks 4 and 12 for assessment of the impact of urinary incontinence on their quality of life. The UDI-6 comprises six items related to lower urinary tract symptoms, including irritative (items 1–2), stress-inducing (item 3–4), and obstructive/discomfort-causing (items 5–6) symptoms. The IIQ-7 assesses seven psychometric items, namely physical activity (items 1–2), travel (items 3–4), social relationships (item 5), and emotional health (items 6–7) [15]. Each item of the UDI-6 and IIQ-7 questionnaires is scored on a four-point scale. The mean score of the items is multiplied by 33 to derive a score ranging from 0 to 100. Higher scores indicate more severe symptoms (UDI-6) and a poorer quality of life (IIQ-7) [15].

The primary outcome was the change in the mean daily number of urge urinary incontinence (UUI) episodes from baseline to week 12 of treatment based on the self-evaluated voiding diary. Secondary outcomes included changes in the mean daily numbers of voiding, incontinence, and nocturia episodes from baseline and the likelihood of achieving a > 50% reduction in UUI and incontinence episodes after 12 weeks. Changes in the OABSS, UDI-6, and IIQ-7 scores after 12 weeks were also examined. Safety was assessed according to the incidence of treatment-emergent adverse events (TEAEs) and was classified using the Common Terminology Criteria for Adverse Events (CTCAE) version 5.0, categorizing grades from 1 to 5 as mild/moderate/severe/life-threatening/death.

### Sample size calculation

Currently, there are no previous studies examining the effectiveness of LLLT devices in patients with OAB. Instead, we searched for clinical trials comparing percutaneous tibial nerve stimulation versus sham for reduction of the number of UUIs per day after 12 weeks of use. As a result, a decrease of 2.4 to 4.6 times was observed in the experimental group. Using a median value of 2.8, we hypothesized that the decrease in the number of urge incontinence events per day after 12 weeks of use was expected to be 2.8 times in the experimental group and 1.5 times in the control group. To prove the difference in the decrease in the number of daily urge incontinence reductions per day after 12 weeks of use between these two groups, 60 patients in each group were required to ensure 5% significance at a a power of 80%. Considering the dropout rate of 25%, a minimum total sample size of 160 patients was necessary. The sample size was calculated using the G*power 3.0.10 software.

### Statistical analysis

Differences in patient characteristics were compared between the study groups using Pearson’s chi-squared test or Fisher’s exact test for categorical variables and the independent *t*-test or Mann-Whitney *U* test for continuous variables as appropriate. Differences in outcomes between the different assessment points were examined using the paired *t*-test or Wilcoxon's signed-rank test. Analysis of covariance was used to compare the primary and secondary outcomes between the groups. The Shapiro-Wilk test was used to test the normality of the data. The last observation carried forward method was used to impute missing values. All statistical analyses were performed using SPSS software (version 22.0; IBM Corp., Armonk, NY, USA). All tests were two-tailed, and a *P*-value of < 0.05 was considered statistically significant.

## Results

### Patient disposition and baseline characteristics

The study flowchart is shown in Fig. 1. Of the 165 patients screened for eligibility, three patients who were not eligible and two who withdrew of consent were excluded. A total of 160 were randomly allocated to one of two study groups. Baseline patient characteristics were consistent across the treatment arms, with 78 (48.8%) patients in the LLLT group and 82 (52.2%) in the sham group (Table 1).

**Table 1.** Baseline characteristics of patients with overactive bladder

Variable	LLLT group n = 78	Sham group n = 82	*P* value
Age, years	60.0 ± 9.7	59.1 ± 10.2	0.796
Body mass index	24.8 ± 3.9	23.5 ± 3.3	0.111
Medical history			
Hypertension	16 (20.5)	21 (25.6)	0.445
Diabetes mellitus	6 (7.7)	10 (12.2)	0.343
Other	18 (23.1)	24 (29.3)	0.374
Parity			0.345
0	4 (5.1)	3 (3.7)	
1	8 (10.3)	11 (13.4)	
2	44 (56.4)	54 (65.9)	
≥ 3	22 (28.2)	14 (17.1)	
Menopausal status			0.656
Premenopausal	14 (17.9)	17 (20.7)	
Postmenopausal	64 (82.1)	65 (79.3)	
Residual urine volume, ml	8.9 ± 18.2	11.2 ± 23.4	0.724

LLLT: low-level light therapy

Data are expressed as mean ± standard deviation or *n* (%) unless otherwise specified

### Efficacy

Compared with the sham group, LLLT group showed a statistically significant improvement in the mean number of daily UUI episodes at week 12 (-1.0 ± 1.7 vs. -0.4 ± 2.5, *P* = 0.003; Table 2). Furthermore, patients in the LLLT group were significantly more likely to report a > 50% reduction in the number of UUI episodes after treatment (66.7% vs. 32.9%, *P* < 0.001).

**Table 2.** Efficacy outcomes for the overall sample of patients with overactive bladder

Variable	Analysis population			Change from week 0		
	LLLT group n = 78	Sham group n = 82	*P* value	LLLT group n = 78	Sham group n = 82	*P* value
Urge urinary incontinence, episode no./day						
Week 0	1.5 ± 1.6	2.2 ± 2.7	0.535	-	-	-
Week 4	0.8 ± 1.5	1.3 ± 2.4	0.071	-0.8 ± 1.8	-0.9 ± 2.3	0.472
Week 12	0.5 ± 1.1	1.8 ± 2.7	< 0.001	-1.0 ± 1.7	-0.4 ± 2.5	0.003
≥ 50% reduction at week 12	52 (66.7)	27 (32.9)	< 0.001	-	-	-
Urinary incontinence, episode no./day						
Week 0	1.7 ± 1.8	2.5 ± 3.8	0.800	-	-	-
Week 4	0.9 ± 1.6	1.5 ± 2.6	0.140	-0.8 ± 1.9	-1.1 ± 3.0	0.479
Week 12	0.6 ± 1.1	2.0 ± 2.9	< 0.001	-1.1 ± 1.9	-0.5 ± 2.9	0.002
≥ 50% reduction at week 12	53 (67.9)	29 (35.4)	< 0.001	-	-	-
Voiding count, episode no./day						
Week 0	10.5 ± 3.6	10.4 ± 3.6	0.955	-	-	-
Week 4	9.3 ± 3.2	9.1 ± 2.5	0.648	-1.2 ± 2.1	-1.2 ± 3.2	0.762
Week 12	8.9 ± 3.5	9.1 ± 2.6	0.105	-1.7 ± 2.9	-1.2 ± 3.5	0.442
Nocturia, episode no./day						
Week 0	1.2 ± 0.9	1.5 ± 0.9	0.099	-	-	-
Week 4	1.1 ± 0.8	1.2 ± 0.8	0.644	-0.1 ± 0.7	-0.3 ± 1.0	0.097
Week 12	0.9 ± 0.8	1.1 ± 1.0	0.220	-0.3 ± 0.7	-0.3 ± 1.0	0.636

LLLT: low-level light therapy

Data are expressed as mean ± standard deviation

Regarding secondary outcomes, there was a significantly greater reduction in the mean number of daily incontinence episodes at week 12 in the LLLT group than in the sham group (-1.1 ± 1.9 vs. -0.5 ± 2.9, *P* = 0.002). Similarly, patients in the LLLT group were significantly more likely to report a > 50% reduction in the frequency of incontinence episodes (67.9% vs. 35.4%, *P* < 0.001). However, at 12 weeks, there was no significant difference in the mean change in the numbers of daily voiding (-1.7 ± 2.9 vs. -1.2 ± 3.5, *P* = 0.442) and nocturia (-0.3 ± 0.7 vs. -0.3 ± 1.0, *P* = 0.636) episodes between the LLLT and sham groups.

There was a decrease in OABSS, UDI-6, and IIQ-7 scores in both study groups, indicating improvement in symptom severity (Table 3). The UDI-6 scores at 12 weeks were significantly lower in the LLLT group than in the sham group (UDI-6, 22.6 ± 15.4 vs. 29.6 ± 18.4, *P* = 0.009). However, there was no significant between-group difference in the mean change in any of the three questionnaire scores between baseline and the final visit.

**Table 3.** Efficacy questionnaire outcomes for patients with overactive bladder

Variable	Analysis population			Change from week 0		
	LLLT group n = 78	Sham group n = 82	*P* value	LLLT group n = 81	Sham group n = 88	*P* value
OABSS total						
Week 0	7.7± 2.3	8.0 ± 2.7	0.650	-	-	-
Week 4	5.9 ± 2.7	6.3 ± 3.0	0.472	-1.8 ± 2.5	-1.6 ± 3.1	0.964
Week 12	5.4 ± 2.9	6.2 ± 3.2	0.179	-2.4 ± 2.8	-1.7 ± 3.4	0.315
UDI-6 score						
Week 0	36.1 ± 16.3	36.8 ± 20.0	0.366	-	-	-
Week 4	26.8 ± 15.9	31.7 ± 19.4	0.099	-9.5 ± 16.4	-8.2 ± 16.5	0.698
Week 12	22.6 ± 15.4	29.6 ± 18.4	0.009	-13.7 ± 18.6	-10.4 ± 17.8	0.248
IIQ-7 score						
Week 0	34.1 ± 19.3	33.8 ± 22.9	0.880	-	-	-
Week 4	24.6 ± 19.3	27.4 ± 19.6	0.306	-9.6 ± 16.3	-6.4 ± 16.6	0.327
Week 12	20.8 ± 19.0	26.0 ± 21.0	0.130	-13.3 ± 16.5	-7.8 ± 16.2	0.103

LLLT: low-level light therapy, OABSS: Overactive Bladder Symptom Score, UDI-6: Urogenital Distress Inventory-6, IIQ-7: Impact Urinary Incontinence-7

Data are expressed as mean ± standard deviation

### Safety and tolerability

The incidence of device-related TEAEs was similar between the LLLT and sham groups (25.9% vs. 25.6%). The device-related TEAEs were maculopapular rash, burning sensation, pigmentation, ulticaria, and pruritus. However, there were no serious TEAEs, such as intolerable abdominal pain or device-related skin reactions. Treatment with the LLLT device was tolerated by all patients (Table 4).

**Table 4.** Treatment-emergent adverse events among patients with overactive bladder

Variable	LLLT group n = 78	Sham group n = 82	*P* value
Skin reaction			
Grade 1	15 (19.2)	17 (20.7)	0.812
Grade 2	5 (6.4)	3 (3.7)	0.487
Abdominal pain			
Grade 1	5 (6.4)	5 (6.1)	0.935
Grade 2	3 (3.8)	0 (0.0)	0.114
Total TEAE	23 (29.5)	25 (30.5)	0.890
Device-related TEAE	20 (25.6)	21 (25.6)	0.996

LLLT: low-level light therapy, TEAE: treatment-emergent adverse event

Data are expressed as *n* (%)

## Discussion

In this randomized comparative study, we evaluated the impact of skin-adhesive LLLT on symptoms of OAB caused by muscle contraction in the wall of the urinary bladder. In patients treated with the LLLT device for 12 weeks, there was a statistically significant reduction in the number of UUIs and incontinence episodes, which are the key symptoms of OAB, comparrd with the sham group. However, there was no significant reduction in voiding or nocturia episodes in either study group. The total UDI-6 questionnaire score was significantly better in the LLLT group than in the sham group after 12 weeks; however, there was no significant between-group difference in the OABSS and IIQ-7 scores at the end of the study. There are several possible explanations for these findings. First, there were marked positive effects in patients who used the sham device, which was unexpected. There is some evidence in the literature suggesting that a sham device can have a beneficial effect [16]. In our study, we applied the LLLT and sham devices at the same acupuncture points, so it is possible that the sham device had a therapeutic effect, which may explain the lack of significant differences in effect between our LLLT and sham groups. Second, our patients had OABSS, UDI-6, and IIQ-7 questionnaire scores at baseline that may have been too low to show significant improvement. Finally, we did not consider potential confounding factors, such as education level, smoking, diet, alcohol and caffeine consumption, and other lifestyle factors known to influence OAB [17]. Although there are already numerous treatment options for OAB, persistence and adherence with treatment continue to be a challenge. A significant proportion of patients did not respond to first-line behavioral therapy and responded poorly, developed unacceptable side effects, or had contraindications to pharmacologic agents, which are used as second-line therapy [18]. Third-line treatments, such as peripheral tibial nerve stimulation, sacral neuromodulation, and intradetrusor injections of onabotulinum toxin A, are available for patients with refractory OAB [18]. While these neuromodulation therapies are known to be more effective in managing OAB, only about 2% of patients receive these treatments [19–21]. LLLT devices may afford an opportunity for effective home-based treatment as a part of a variety of approaches to treating OAB. Moreover, these devices may overcome the problem of poor adherence because any adverse effects are tolerable and they can be used easily without interfering with everyday activities, such as watching television, exercising, and doing housework.

The two acupuncture treatment points applied in our study are commonly used to manage OAB in traditional Chinese/Korean medicine [14]. CV6 is called Qihai, the Sea of Qi, located 1.5 cun inferior to the umbilicus, and CV4 is called Guanyuan, Gate of Origin, located 3 cun inferior to the umbilicus [9, 22]. Both acupuncture points are thought to reduce OAB symptoms because they are major targeting points for female urology. According to previous randomized controlled trials, acupuncture was confirmed to be effective in treating OAB [23, 24]. However, the exact mechanisms and function of targeting these acupuncture points remain unclear.

This study has some limitations. First, as in other randomized studies performed in patients with OAB, we assessed the efficacy and safety of using the LLLT device when used for 12 weeks, which may have been a little short to find a significant reduction in symptoms of LLLT. This may be why the absolute differences between groups are low and have relative importance. Second, we did not measure the levels of light-induced substances, such as cyclic adenosine monophosphate and NO. However, our findings are consistent with the results of previous studies that have demonstrated relaxation of contracted or weakened muscle tissue in the wall of the urinary bladder in response to LLLT at acupuncture points. Third, as we mentioned above, we did not consider potential confounding factors that could influence our results and cause some bias. Finally, there was a high dropout rate, which limits the statistical value of our data. Most of the dropouts were because the patients felt the burden of filling out various forms such as voiding diary and questionnaires.

In conclusion, to our knowledge, this randomized, placebo-controlled clinical trial is the first to evaluate the efficacy and safety of skin-adhesive LLLT administered to acupuncture points in patients with OAB. In this study, application of skin-adhesive LLLT to acupuncture points for 12 weeks was a safe and effective treatment for OAB. The LLLT device may be an excellent non-pharmacologic treatment option for patients with OAB and have an impact on clinical management.
